# Deep Brain Stimulation of the Subthalamic Nucleus Modulates Reward-Related Behavior: A Systematic Review

**DOI:** 10.3389/fnhum.2020.578564

**Published:** 2020-11-20

**Authors:** Yvan M. Vachez, Meaghan C. Creed

**Affiliations:** ^1^Department of Anesthesiology, Washington University Pain Center, Washington University School of Medicine, St. Louis, MO, United States; ^2^Departments of Psychiatry, Neuroscience and Biomedical Engineering, Washington University School of Medicine, St. Louis, MO, United States

**Keywords:** deep brain stimulation (DBS), subthalamic nucleus (STN), reward, motivation, rodent, operant

## Abstract

Deep brain stimulation of the subthalamic nucleus (STN-DBS) is an effective treatment for the motor symptoms of movement disorders including Parkinson's Disease (PD). Despite its therapeutic benefits, STN-DBS has been associated with adverse effects on mood and cognition. Specifically, apathy, which is defined as a loss of motivation, has been reported to emerge or to worsen following STN-DBS. However, it is often challenging to disentangle the effects of STN-DBS *per se* from concurrent reduction of dopamine replacement therapy, from underlying PD pathology or from disease progression. To this end, pre-clinical models allow for the dissociation of each of these factors, and to establish neural substrates underlying the emergence of motivational symptoms following STN-DBS. Here, we performed a systematic analysis of rodent studies assessing the effects of STN-DBS on reward seeking, reward motivation and reward consumption across a variety of behavioral paradigms. We find that STN-DBS decreases reward seeking in the majority of experiments, and we outline how design of the behavioral task and DBS parameters can influence experimental outcomes. While an early hypothesis posited that DBS acts as a “functional lesion,” an analysis of lesions and inhibition of the STN revealed no consistent pattern on reward-related behavior. Thus, we discuss alternative mechanisms that could contribute to the amotivational effects of STN-DBS. We also argue that optogenetic-assisted circuit dissection could yield important insight into the effects of the STN on motivated behavior in health and disease. Understanding the mechanisms underlying the effects of STN-DBS on motivated behavior-will be critical for optimizing the clinical application of STN-DBS.

## Introduction

Deep brain stimulation (DBS) is a surgical therapy whereby electric current is passed through electrodes implanted into specific brain nuclei. DBS applied to the subthalamic nucleus (STN-DBS) has been extensively used to treat motor symptoms of Parkinson Disease (PD) for more than 30 years (Benabid et al., 2009). This neurosurgical treatment is typically applied in patients after years of first-line dopamine replacement therapy (i.e., L-DOPA), which eventually loses its efficacy and starts to induce dyskinesias which further reduce its therapeutic utility (Poewe, 1994; Ahlskog and Muenter, 2001; Obeso et al., 2004). STN-DBS significantly improves PD motor symptoms of tremor, rigidity and akinesia (Limousin et al., 1995; Krack et al., 2003; Fasano et al., 2010) and thus reduces the required dose of dopaminergic agonist or replacement therapy (Moro et al., 1999). Because of its reliable therapeutic efficacy, it has been proposed to apply STN-DBS earlier in the course of PD, before dopaminergic therapy loses efficacy or the emergence of L-Dopa induced dyskinesias (Deuschl et al., 2013; Schuepbach et al., 2013). Moreover, case reports have suggested that STN-DBS may reduce compulsive (Mallet et al., 2002; Fontaine et al., 2004) or addiction-like behaviors (Witjas et al., 2005), which has led to the suggestion that STN-DBS could be applied in patients suffering from obsessive compulsive disorder (Mallet et al., 2008) or to reduce symptoms of substance use disorders (Krack et al., 2010; Rouaud et al., 2010; Pelloux and Baunez, 2013). As a result of earlier intervention with STN-DBS for PD, as well as the increasing indications, the population of patients treated with STN-DBS will expand to more heterogeneous populations.

Along with its therapeutic benefits, neuropsychiatric side effects of STN-DBS have been reported since its first applications. Reported effects range from new onset or worsening of impulsivity, apathy or anhedonia to improvement of pre-existing behavioral symptoms (Chaudhuri and Schapira, 2009; Castrioto et al., 2014). Dissociating the effects of STN-DBS itself from underlying neuropathology and co-occurring pharmacological treatment is critical to understand the etiology of these side effects. One of the most frequently reported side effects of STN-DBS in the clinic is apathy, defined as a loss of motivation or reduction in goal-directed behavior accompanied by flattened affect (Marin, 1996; Levy and Dubois, 2006). Apathy is a core neuropsychiatric symptom of PD, that can be present before STN-DBS and alleviated by dopaminergic agonists (Leentjens et al., 2009). Apathy can be exacerbated following STN-DBS (Drapier et al., 2006; Le Jeune et al., 2009; Wang et al., 2018), which then compromises the quality of life benefits of STN-DBS (Maier et al., 2013, 2016; Martinez-Fernandez et al., 2016). The prevailing explanation for the emergence or worsening of apathy following STN-DBS is a withdrawal-like syndrome due to the reduction of dopaminergic treatment (Thobois et al., 2010; Chagraoui et al., 2018), although there is also evidence supporting a role for STN-DBS itself in this pathogenesis (Le Jeune et al., 2009; Zoon et al., 2019). However, because of the interaction between STN-DBS, pharmacological co-treatments and the progression of PD pathology, it is difficult to determine the underlying causes of motivational symptoms arising after STN-DBS in the clinic.

In this respect, pre-clinical models have the advantage of being able to isolate the contribution of STN-DBS alone to motivation-related behaviors, and to elucidate the neural mechanisms underlying these behaviors. To date, several studies have sought to determine the involvement of STN modulation on motivational processes (for reviews see Temel et al., 2009; Baunez and Gubellini, 2010; Hamani et al., 2017). However, several methodological differences exist between these studies, including the behavioral paradigm used to assess motivation, the parameters of stimulation or the reward used, which has precluded any clear consensus regarding the effects of STN-DBS on motivation and reward processing. To address this controversy, we performed a systematic review of the pre-clinical literature, extracting features of studies focused specifically on reward motivation and consumption behaviors. We identify a consistent pattern of decreased reward seeking, motivation and consumption induced by STN-DBS, which was not evident in studies of STN lesion or inactivation. We also identify several stimulation and experimental parameters that are associated with STN-DBS-induced motivational deficits. Our analysis provides a rationale for using pre-clinical models to dissect the neural mechanisms underlying specific behavioral effects of STN-DBS. This mechanistic understanding will be critical for optimizing STN-DBS as it is applied to expanding patient populations and for increasing clinical indications.

## Methods

We systematically analyzed all pre-clinical studies investigating the effect of STN-DBS, STN Lesion, or pharmacological inhibition of the STN on motivation for reward.

### Identification of Pertinent Literature

A systematic analysis of the international literature was carried out by selecting articles published in peer-review journals, using PubMed, and BioRxiv databases. The last search was conducted on September 11, 2020. Restrictions were made, limiting the study to academic publications in which the full text was published in English. Search terms were as follows: “subthalamic nucleus” AND “stimulation” AND (“reward” OR “motivation” OR “self-administration” OR “addiction” OR “cocaine” OR “FOOD”); and “subthalamic nucleus” AND (“inactivation” OR “lesion”).

### Screening and Eligibility

**Figure 1:**
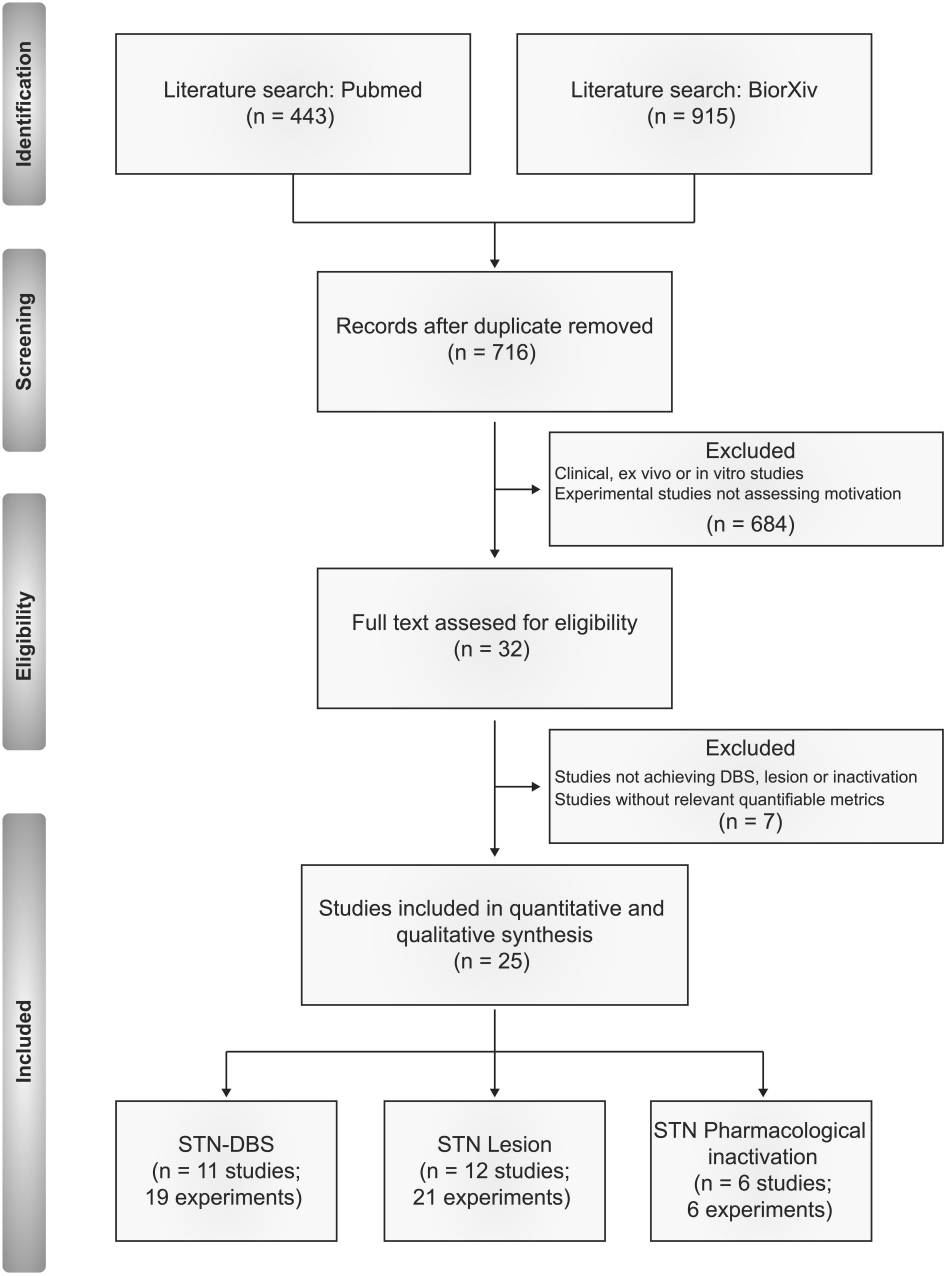
PRISMA flow diagram of the inclusion criteria of studies eligible for systematic review.

From the list of potential articles produced by systematic research, we selected studies relevant to the topic on the basis of their title and abstract. In brief, we excluded clinical, *in vitro* and *ex vivo* investigations, along with experimental studies on rodents not assessing motivation or reward-related behaviors. We then excluded studies applying neuromodulation techniques other than electrical stimulation, lesion or pharmacological inactivation, or studies not providing metrics relevant to the criteria outlined below (Figure 1).

### Studies Included

**Figure 2:**
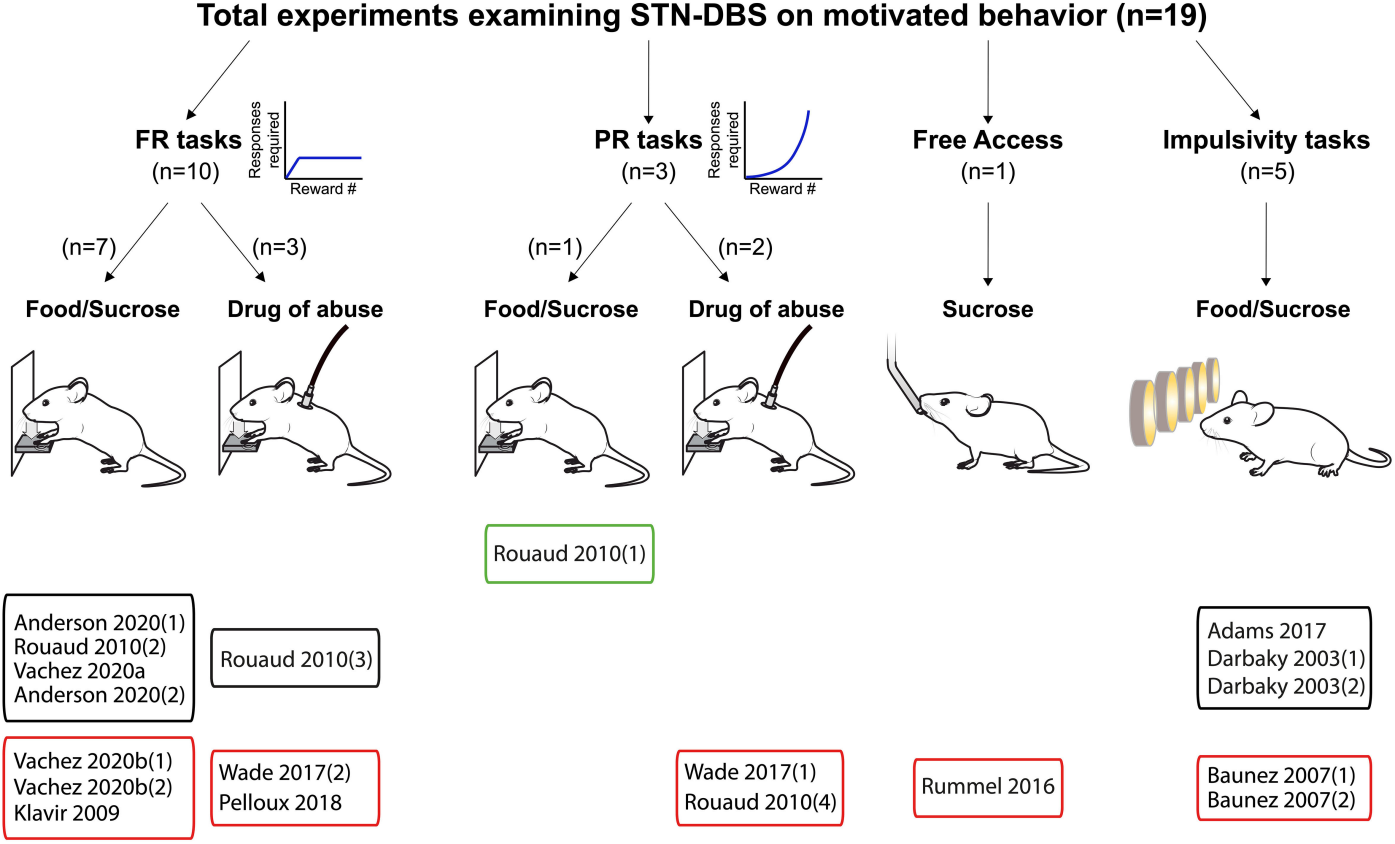
Experiments investigating STN-DBS effects on reward-related behavior. Graphical representation of experiments assessing the effects of STN-DBS on reward-related behavior. Experiments are split according the task and the reward provided, and whether the main effect was an increase (green frames), decrease (red frames) or no change (black frames) in motivational state induced by STN-DBS. FR, Fixed ratio; PR, progressive ratio.

**Figure 3:**
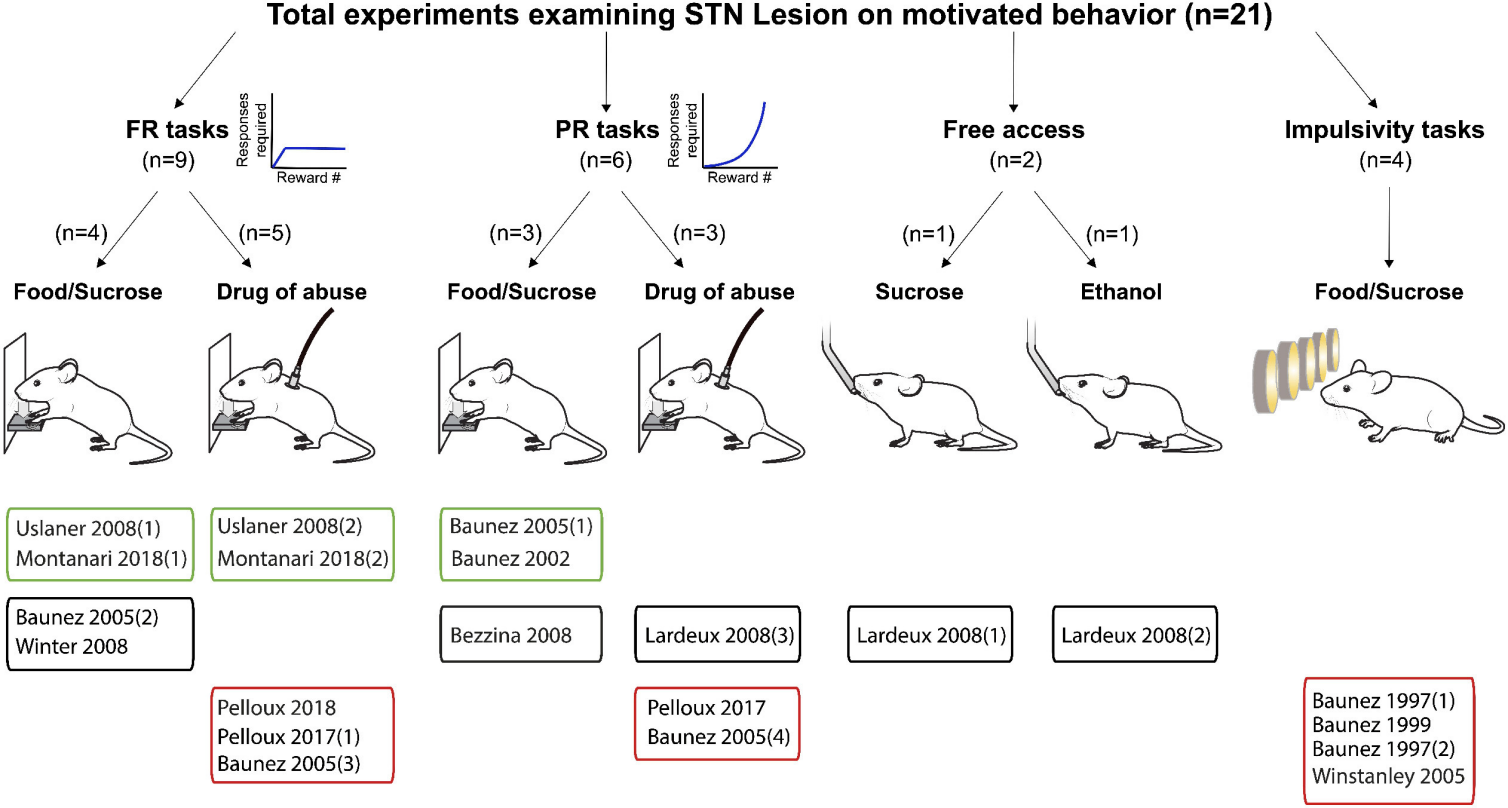
Experiments investigating STN lesion effects on reward related behavior. Graphical representation of experiments assessing the effects of STN lesion on reward-related behavior. Experiments are split according the task, the reward provided and whether the main effect was an increase (green frames), decrease (red frames) or no change (black frames) in motivational state following lesion of the STN. FR, Fixed ratio; PR, progressive ratio.

**Figure 4:**
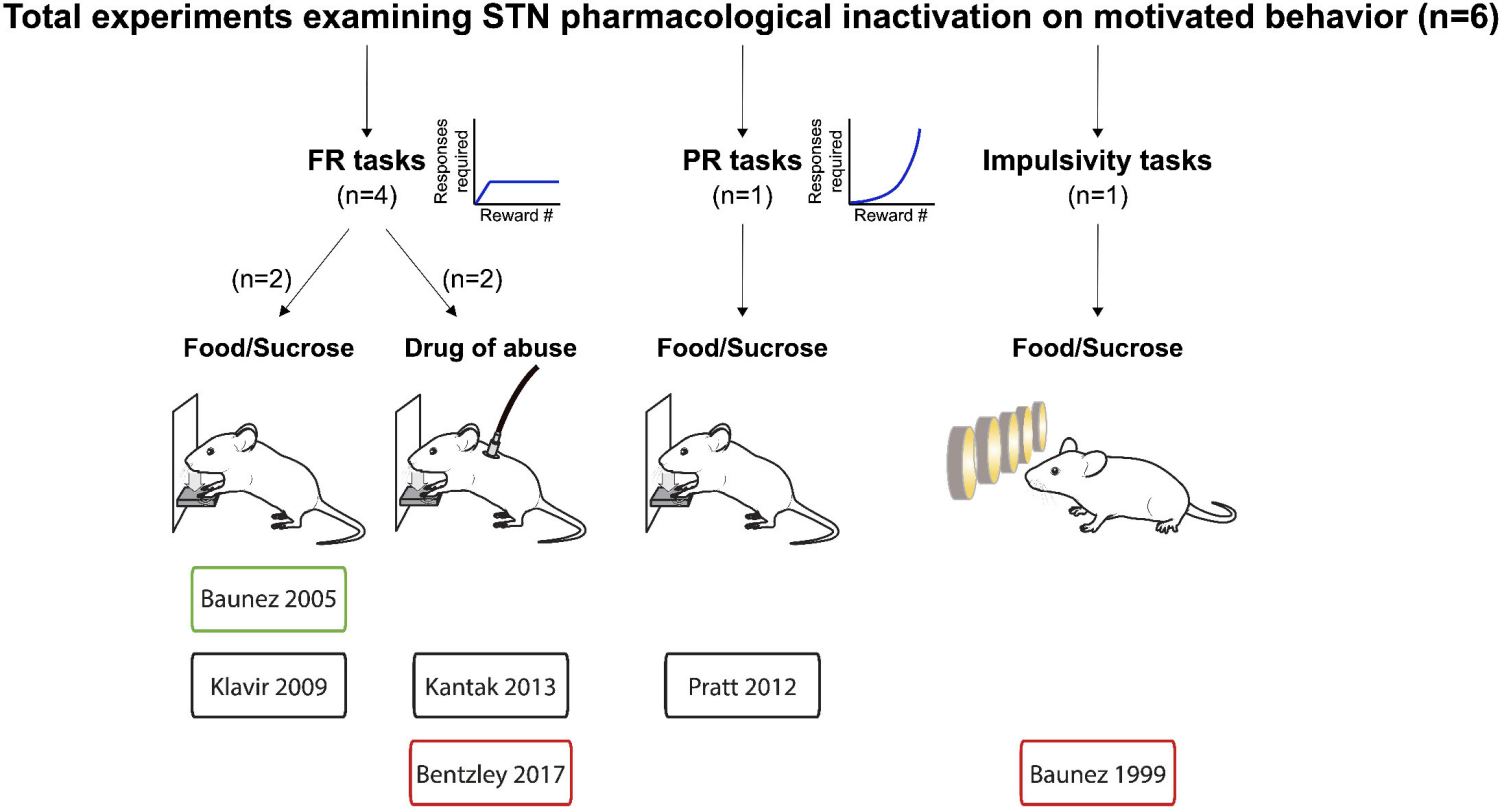
Experiments investigating STN pharmacological inhibition effects on reward related behavior. Graphical representation of experiments assessing the effects of pharmacological inactivation of the STN on reward-related behavior. Experiments are split according the task, the reward provided and whether the main effect was an increase (green frames), decrease (red frames) or no change (black frames) in motivational state following pharmacological inactivation of the STN. FR, Fixed ratio; PR, progressive ratio.

Following this approach, we included 46 relevant experiments across 25 published studies between 1997 and 2020. We summarize the composition of these studies in Figures 2–4.

In rodents, assessment of motivation often relies on reward seeking tasks, during which the animal has to perform an operant behavior to receive a reward (Koob and Weiss, 1990). The majority (33/46) of the experiments in our analysis used a standard operant reinforcement task consisting of lever pressing or nose poke to induce reward delivery. Twenty two of the 33 studies used a fixed ratio (FR) paradigm, in which a fixed number of operant responses (lever press or nose poke) is necessary to earn a reward. Most studies (19) used a FR1 paradigm; here, we extracted the number of operant responses and earned rewards to evaluate motivational changes. The remaining 11 operant experiments used the progressive ratio (PR) task, in which the number of required operant responses increases incrementally with each reward earned during the task (Arnold and Roberts, 1997; Bradshaw and Killeen, 2012). The PR task is used to assess motivation by establishing “break-point,” or the number of operant responses the animal is willing to execute in order to obtain the reward (Griffiths et al., 1975). We extracted the break-point, or, if not available, the number of rewards earned. Few additional experiments (*n* = 3) provided the reward in a free access task, which require no explicit amount of work to obtain a reward. We thus use the consumed reward quantity as an outcome measure.

Finally, we included ten experiments that use tasks designed to assess impulsive behavior in the context of reward seeking. The majority of these studies (*n* = 8) used variations of the five-choice serial-reaction time task (5-CSRTT) (Robbins, 2002), while single studies using a delay discounting task (Evenden and Ryan, 1996) and rat Iowa Gambling Task (rIGT; van den Bos et al., 2014). In each of these paradigms, the start of the trial is cued, and the animal is required make the choice to complete a trial or not, and to consume the reward if the trial was successful.

The primary outcome of these tasks is to assess impulsivity by using metrics such as pre-mature responses. However, several additional parameters such as the number of non-completed trials (omissions), failures to retrieve the reward, degree of perseverative responding or the latency to execute the operant behavior or reward retrieval can be gleaned from these tasks. Changes in these parameters can reflect altered cognitive processing, motor impairments, attentional deficits or motivational changes. Motivational changes can be inferred with caution by the evolution of the numbers of omissions, especially when coupled with an increase in response latency (Robbins, 2002; Higgins and Silenieks, 2017). In few occasions, perseverative responses have been interpreted as reflecting changes in motivation (Baunez and Robbins, 1997; Baunez et al., 2007), although they are more frequently interpreted as evidence for compulsive behaviors rather than motivation *per se* (Robbins, 2002; Higgins and Silenieks, 2017). Thus, in order to extract the motivational components of the task of these different studies in a comparable and consistent way, we limited our analysis to the quantification of reward omissions.

The studies in our analysis also varied in terms of type of reward. In 29 of 45 experiments, the delivered reward was palatable food, generally sucrose pellets or solution. While in the remaining 16 experiments, a drug reward (cocaine, heroin or ethanol) was used.

**Figure 5:**
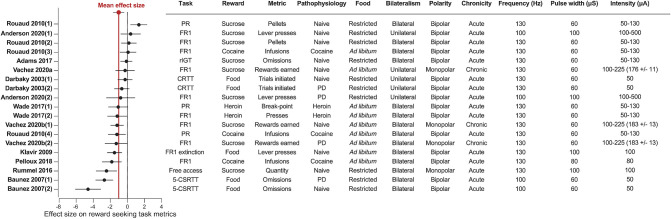
STN-DBS decreases reward related behavior. Forest plot of the Cohen's d standardized mean difference for reward seeking effect of STN-DBS. Mean effect size is depicted by the dashed red line. Key details of the experimental design, and DBS stimulation parameters are summarized in the associated table. All the studies provided water *ad libitum*. 5-CSRTT, Five choice serial reaction time; CRTT, choice reaction time task; PD, Parkinson's disease; FR, Fixed ratio; PR, progressive ratio; rIGT, rat Iowa Gambling Task.

**Figure 6:**
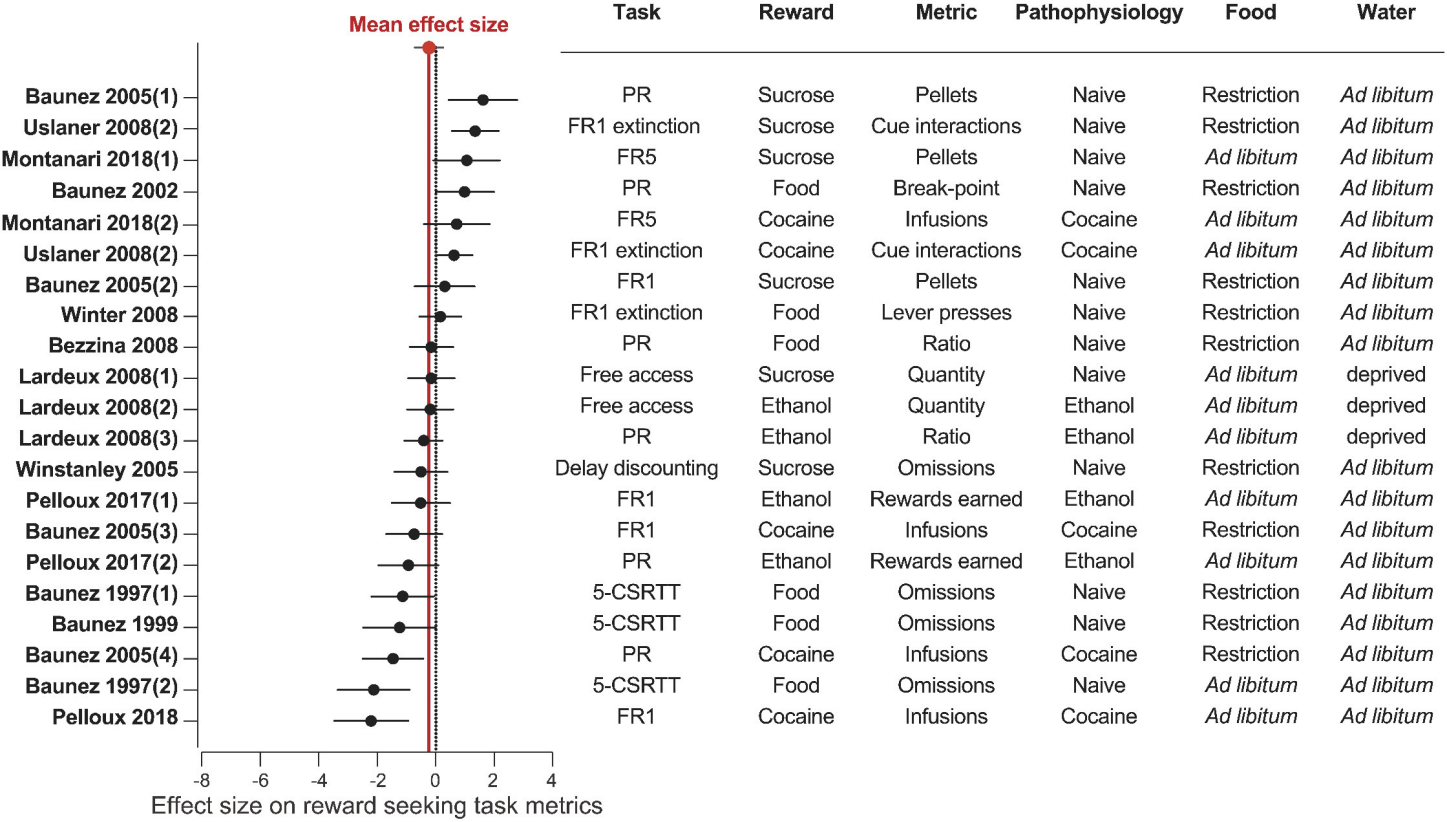
STN lesion does not consistently affect reward related behavior. Forest plot of the Cohen's d standardized mean difference for reward seeking effect of STN lesion, ranked in order of positive to negative effect. Mean effect size is depicted by the dashed red line. 5-CSRTT, five choice serial reaction time; FR, Fixed ratio; PR, progressive ratio.

**Figure 7:**
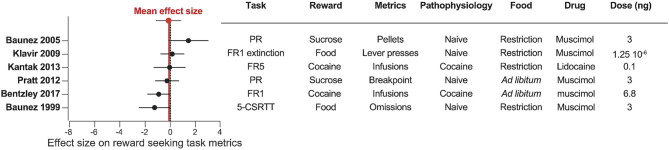
Pharmacological inactivation of the STN does not consistently affect reward related behavior. Forest plot of the Cohen's d standardized mean difference for reward seeking effect of STN pharmacological inactivation, ranked in order of positive to negative effect. Mean effect size is depicted by the dashed red line. 5-CSRTT, five choice serial reaction time test; FR, fixed ratio; PR, progressive ratio.

When precise metrics were not provided in the results description, means and SEM were extracted from graphical results section using Engauge Digitizer 12.1 software. For each experiment we calculated the Cohen's d standardized mean difference (mean difference divided by the pooled standard deviation) as an estimate of the effect size (Lee, 2016). Thus, we excluded studies if data were not shown or if the precise number of animals for each experimental group was not provided. We represented each effect size ± 95% confidence intervals on forest plots (Figures 5–7).

## Results

Our results reveal a consistent effect of STN-DBS decreasing reward motivation and consumption (Figures 2, 5). These results also highlight specific experimental factors related to task design or stimulation parameters that may influence the magnitude of STN-DBS effect on reward-related behavior. Finally, while early hypotheses posited that STN-DBS induces a “functional lesion” of the STN through inactivation via depolarization block (Beurrier et al., 2001; Magarinos-Ascone et al., 2002; Jakobs et al., 2019), our analysis indicates that this decrease in reward seeking is not recapitulated by lesioning or pharmacologically inhibiting the STN (Figures 3, 4, 6, 7).

### STN-DBS Decreases Reward Seeking

The systematic analysis of studies using STN-DBS revealed a consistent pattern of decreased reward-seeking, which is summarized in Figures 2, 5. In fact, only a single study reported an increase (30%) in motivation for reward measured by the number of sucrose pellets earned during a PR task (Rouaud et al., 2010). The majority of investigations reported a significant decrease in reward motivation (10/19), while a smaller number found no effect (8/19) (Darbaky et al., 2003; Rouaud et al., 2010; Anderson et al., 2020). In FR or PR operant tasks, STN-DBS consistently decreased intravenous self-administration of addictive drugs (Rouaud et al., 2010; Wade et al., 2017). And while the effects of STN-DBS on motivation for natural rewards is more heterogeneous, the predominant effect of STN-DBS is also a decrease in rewards earned and consumed (Rummel et al., 2016) (Vachez et al., 2020b), but see (Rouaud et al., 2010; Vachez et al., 2020a). A decrease in reward seeking was also evident in extinction tasks, i.e., where STN-DBS operant responses were decreased in the absence of a previously available food pellet (Klavir et al., 2009). Finally, STN-DBS increased the rate of trial omissions in impulsivity tasks (Baunez et al., 2007; Adams et al., 2017), which is one index of decreased reward motivation (Robbins, 2002). In summary, the predominant effect of STN-DBS across these tasks is a reduction in reward seeking and motivation, measured by decreased operant responses, rewards earned, rewards consumed or increased trial omissions. In the following section, we discuss factors that contribute to the variance in results found between studies, which are important to consider when assessing the translational impact of these findings.

### Acute vs. Chronic DBS

When interpreting the clinical relevance of STN-DBS in experimental models, one has to keep in mind that patients are stimulated chronically, continuously and that prolonged STN-DBS can drive long term plasticity within the STN or its target nuclei (Shen et al., 2003; Lavian et al., 2013; Chassain et al., 2016). In patients, some therapeutic motor effects of STN-DBS, such as tremor cessation, appear immediately, while it can take several weeks for other symptoms, such as postural instability, to improve (Herrington et al., 2016). The same acute vs. chronic distinction can be made regarding neuropsychiatric symptoms. Some symptoms occur immediately upon STN-DBS onset, such as hypomania, laughing or crying (Krack et al., 2001; Mallet et al., 2007; Wojtecki et al., 2007; Abulseoud et al., 2016), while other symptoms, typically apathy, progressively emerge with chronic stimulation (Drapier et al., 2006; Le Jeune et al., 2009).

In our analysis, only three experiments applied STN-DBS chronically, and two of these experiments showed a significant decrease of sucrose or food self-administration over time (Vachez et al., 2020b). This is in contrast with the absence of motivational deficits during acute STN-DBS during a similar FR1 task or even the increased motivation during a PR task (Rouaud et al., 2010). These differential effects could suggest potential long-term adaptations in the mesolimbic system underlying motivational deficit following chronic STN-DBS. Further investigations specifically using chronic STN-DBS (Melon et al., 2015; Chassain et al., 2016) are needed to understand the long-term effect of STN-DBS on reward-related behavior, and potential plasticity mechanisms underlying these behavioral adaptations.

### Unilateral vs. Bilateral DBS

Another important factor to consider when interpreting the effects of STN-DBS on reward seeking is whether the stimulation is applied unilaterally (to a single hemisphere) or bilaterally. One set of studies performed under matched conditions from the same group reported that chronic unilateral STN-DBS during a FR1 task (Vachez et al., 2020a) did not recapitulate the sustained reward seeking deficit that occurred with chronic, bilateral stimulation (Vachez et al., 2020b). With the unilateral STN-DBS, the effect was only transient and lasted no more than 5 days. Overall, the few studies using unilateral STN-DBS do not report robust reward seeking deficits, or report deficits that are only transient (Darbaky et al., 2003; Anderson et al., 2020; Vachez et al., 2020a). In the clinic, bilateral STN-DBS is generally associated with superior reduction in motor symptoms relative to unilateral stimulation (Bastian et al., 2003; Lizarraga et al., 2016), but may also induce more non-motor side-effects (Lee et al., 2011; Sjoberg et al., 2012). Notably, a study within the same clinical center observed apathy following bilateral (Le Jeune et al., 2009) but not unilateral STN-DBS (Vachez et al., 2020a). These observations suggest that preserving function of one STN by applying DBS unilaterally protects against a reward seeking deficits in patients or in animal models. It is also possible that DBS may drive compensatory metabolic or neural circuit changes in the un-stimulated hemisphere that may mitigate reward-seeking deficits induced by STN-DBS. Thus, whether STN-DBS is applied uni- or bilaterally is an important factor to consider when interpreting STN-DBS effects in animal models and its relevance to clinical populations.

### Stimulation Polarity

In patients, monopolar electrodes are preferentially used, with the pulse generator within the chest being the ground pole of stimulation (Benabid et al., 2009; Amon and Alesch, 2017). In contrast, most rodent studies use bipolar electrodes. Bipolar stimulation generates a more focal electric field than monopolar electrodes and consequently activates a smaller volume of tissue (Temel et al., 2004; Chaturvedi et al., 2013; Hancu et al., 2019). While there are very few published direct comparisons of bipolar and monopolar stimulation within patients, monopolar stimulation is associated with greater improvement of rigidity, tremor and bradykinesia, but also with a higher incidence of side-effects such as confusion or mania (Deli et al., 2011; Chopra et al., 2012). We found a single study that directly compared monopolar and bipolar STN-DBS (Badstuebner et al., 2017). Consistent with clinical observations, monopolar STN-DBS was also associated with a greater reduction in akinesia, sensorimotor neglect and amphetamine-induced rotation than bipolar DBS in 6-OHDA lesioned rats (Badstuebner et al., 2017). However, monopolar STN-DBS is rarely used in rodent studies, so it is difficult to draw firm conclusions regarding the difference between monopolar and bipolar stimulation on reward-related behavior. Interestingly, the only studies that reported STN-DBS-induced reward seeking deficits in low effort tasks (FR1 or free access consumption) used monopolar electrodes (Rummel et al., 2016; Vachez et al., 2020b). This potential greater decrease in reward motivation with monopolar STN-DBS could be explained by differences in current spread. As we will discuss in subsequent section, although the motor territory of the STN is targeted with DBS, electric current can feasibly spread to associative or limbic territories, or even to adjacent neural structures (Mandat et al., 2006; Tan et al., 2013). Because the electric field induced by monopolar stimulation is more diffuse than with bipolar stimulation, this current spread could be an important driver of decreased reward seeking and motivation.

### Pathophysiological and Metabolic State

Finally, the underlying pathophysiology and metabolic state of animals must be carefully considered when interpreting effects of STN-DBS on reward-related behavior. Although STN-DBS has primarily been studied in the context of PD, few studies have directly examined the effects of STN-DBS on reward seeking and motivation in experimental models of PD. In rodents, PD is typically modeled by the selective ablation of dopaminergic neurons with intracranial injections of 6-hydrodopamine (6-OHDA) to mimic the degeneration of dopaminergic neurons observed in PD (Deumens et al., 2002). Dopamine is critical for encoding reward value, action selection and vigor as well as updating behaviors based on past history of prior rewards and punishments [for review, see Berke (2018)]. Therefore, according to the specificity and extent of the dopaminergic lesion, decreased motivation and operant responding for sucrose frequently occurs in 6-OHDA-lesioned animal models independent of STN-DBS (Drui et al., 2014; Favier et al., 2014, 2017; Magnard et al., 2016). Yet, the characteristic striatal dopaminergic denervation in these PD models does not appear to influence the outcome of STN-DBS reward-related behavior. In intact and in 6-OHDA-lesioned rats, STN-DBS induces a similar rate of omission in choice reaction time task (Darbaky et al., 2003; Baunez et al., 2007) and an equivalent decrease of the number of sucrose rewards earned during a FR1 task (Vachez et al., 2020b).

A related factor that definitely affects motivation and thus outcomes of reward seeking tasks is the baseline satiety state of the animal (Berridge, 2004). Some level of food restriction is commonly used to invigorate seeking behaviors and learning in complex tasks such as the 5-CSRTT, and it does so by increasing the motivational value of the reward (Cabeza de Vaca and Carr, 1998; Mosberger et al., 2016). Thus, it is likely that basal food restriction can account for some of the lack of effect of STN-DBS observed in sucrose self-administration studies under low demand conditions [i.e., FR1, Rouaud et al. (2010) and Anderson et al. (2020)], while experiments with this same FR1 task conducted without food restriction have found decreases in reward seeking (Vachez et al., 2020b). The interpretation is that under conditions of basal food restriction, homeostatic drive for calories in sucrose overrides more subtle effects of STN-DBS; when there is no underlying metabolic demand for sucrose, the effects of STN-DBS on incentive motivation are more readily apparent.

### Food or Drug Reward

A final and related consideration is whether food or drug reward is used to probe the effects of STN-DNS on reward seeking. Whereas 5/14 experiments reported decreased motivation for sucrose or food reward, 4/5 studies using drug reward found that STN-DBS decreases motivation for the drug. Briefly, bilateral STN-DBS decreases on-going self-administration and escalation of drug taking for both cocaine and heroin (Rouaud et al., 2010; Wade et al., 2017; Pelloux et al., 2018) and decreases relapse to heroin seeking following protracted abstinence (Wade et al., 2017). Importantly, STN-DBS has opposite effects in the same investigation according to the reward; decreasing cocaine self-administration but increasing sucrose taking (Rouaud et al., 2010). Some evidences suggest that the STN encodes reward value (Lardeux et al., 2009); and that sucrose and cocaine elicit activity of different subthalamic neuronal populations (Lardeux et al., 2013). Thus, it is hypothesized that different microcircuits within the STN separately drive motivation for “natural” reward or for addictive drugs. It is therefore possible that these microcircuits could be differentially impacted by STN-DBS, which may explain the more consistent effects of STN-DBS on decreasing motivation for drugs of abuse. Additional work is needed to understand how motivation for drugs abuse is encoded within the STN during different phases of the addiction cycle, which will have important implications for optimizing STN-DBS as a potential therapy for substance use disorders.

### Summary

Overall, when applied chronically, bilaterally and with monopolar electrodes to model clinically-relevant conditions, STN-DBS consistently decreases reward seeking behavior. This STN-DBS-induced decrease in reward seeking is consistent across operant tasks but is most evident in satiated rats. Some attempts have been made to harness this feature, by proposing STN-DBS as a potential therapy for addiction (Rouaud et al., 2010; Pelloux and Baunez, 2013; Creed M. C., 2018).

However, this review highlights that STN-DBS has the capacity to decrease seeking for natural rewards as well as for drugs of abuse. A related consideration is that chronic application of STN-DBS leads to the emergence of learned-helplessness behaviors in shuttle-box or forced swim tasks (Temel et al., 2007; Tan et al., 2011; Creed et al., 2013). These results suggest that a general amotivational state may be induced by chronic STN-DBS in rodents, and emphasize the need for a mechanistic understanding of how STN-DBS induces its effects on reward-seeking in order to optimize the therapy for movement or substance-use disorders.

## Determining the Neural Mechanisms Underlying the Effects of STN-DBS

### STN-DBS Is Not Equivalent to Functional Inactivation

One early hypothesis regarding the mechanism of action of DBS is that stimulation silences local cell bodies (Grill et al., 2004; McIntyre and Anderson, 2016), producing a functional lesion. In PD models and patients, lesioning the STN abolishes pathological hyperactivity and burst firing within the nucleus (Bergman et al., 1994; Hassani et al., 1996; Kreiss et al., 1997; Vila et al., 2000) that is correlated with motor symptoms (Bergman et al., 1990; Guridi et al., 1994, 1996; Wichmann et al., 1994; Henderson et al., 1999; Baron et al., 2002; Kühn et al., 2009; Baunez and Gubellini, 2010). Consistent with this silencing mechanism, STN-DBS inhibits firing of local STN neurons both *ex vivo* and *in vivo* (Benazzouz et al., 2000b; Tai et al., 2003; Filali et al., 2004; Welter et al., 2004; Meissner et al., 2005; Shi et al., 2006; Wade et al., 2017). Multiple mechanisms have been proposed to account for this inhibition, including voltage-dependent activation of potassium conductance resulting shunt inhibition (Shin et al., 2007; Florence et al., 2016), inactivation of sodium channels (Beurrier et al., 2001; Magarinos-Ascone et al., 2002) neuronal energy depletion (Lozano et al., 2002) or excitation of pallidal GABAergic terminals to the STN (Filali et al., 2004). While a functional lesion effect has been proposed to account for many of the motor effects of DBS, whether this functional silencing also accounts for adverse psychiatric effects of STN-DBS is less clear. To address this question, we analyzed pre-clinical studies that examined the effect of either electrolytic lesion or pharmacological inactivation of the STN on reward seeking. This inactivation was achieved with muscimol (an agonist of the GABAA receptor which fluxes chloride ions into the cell, thereby hyperpolarizing the membrane) or lidocaine (an antagonist of voltage-gated sodium channels, which are required for action potential firing).

Results of STN lesion studies were more heterogeneous than with DBS, with increases (*n* = 6), decreases (*n* = 9), or no change in reward seeking (*n* = 6) being reported (Figures 3, 6). This heterogeneity cannot be completely explained by the type of reward used; STN-lesions had heterogeneous effects on responding for sucrose and food (Baunez and Robbins, 1997, 1999a; Baunez et al., 2002, 2005; Winstanley et al., 2005; Bezzina et al., 2008; Lardeux and Baunez, 2008; Uslaner et al., 2008; Winter et al., 2008), as well as for addictive drugs (Baunez et al., 2005; Lardeux and Baunez, 2008; Uslaner et al., 2008; Pelloux and Baunez, 2017; Montanari et al., 2018; Pelloux et al., 2018).

The heterogeneity could partially be explained by the behavioral paradigm used. STN lesion did not affect reward intake in free access (Lardeux and Baunez, 2008) or extinction paradigms (Winter et al., 2008) where the cost of responding is low. In impulsivity tasks, STN lesion consistently increased the rate of trial omissions and response latency (Baunez and Robbins, 1997, 1999a; Winstanley et al., 2005), which can be interpreted as decreased motivation (Robbins, 2002; Higgins and Silenieks, 2017) (Figures 3, 6). However, when assayed using classical FR or PR operant tasks, STN lesion either increased (Baunez et al., 2002, 2005; Uslaner et al., 2008; Montanari et al., 2018) or did not change (Baunez et al., 2005; Bezzina et al., 2008; Winter et al., 2008) self-administration of food or sucrose. Decreased cocaine or ethanol taking is the predominantly reported effect of STN lesion (Baunez et al., 2005; Pelloux and Baunez, 2017; Pelloux et al., 2018). However, absence of effect (Bezzina et al., 2008) and even slightly increased drug seeking (Uslaner et al., 2008; Montanari et al., 2018) has also been reported following STN lesion.

Fewer studies have investigated pharmacological inactivation of the STN (Figures 4, 7). Of the six total studies, one experiment reported an increase of food pellets earned during an operant task (Baunez et al., 2005), while three studies reported no effect on food or sucrose pellets (Klavir et al., 2009; Pratt et al., 2012) or cocaine administration (Kantak et al., 2013). Finally, two experiments showed decreased reward seeking with STN inactivation, measured as increased omissions in a food-rewarded 5-CSRTT (Baunez and Robbins, 1999b; Bentzley and Aston-Jones, 2017) or reduced cocaine self-administration (Bentzley and Aston-Jones, 2017). Overall, the mean effect size of STN lesion or inhibition is null, owing to the high variability in experimental outcomes reflecting the heterogeneity of the experimental conditions in terms of task, reward, physiological and metabolic state. These results are in stark contrast to the consistent effects of STN-DBS across studies, and suggest that STN-DBS effects cannot be emulated with lesion or pharmacological inactivation.

### STN-DBS Modulates Activity Throughout the Basal Ganglia

The difference in behavioral outcomes between STN-DBS and STN-lesion and inactivation is not entirely surprising. While the predominant effect of DBS on local tissue is depolarization block (Benazzouz et al., 1995; Beurrier et al., 2001; Tai et al., 2003), DBS can modulate distal brain regions through antidromic and orthodromic activation (Fedele and Raiteri, 1999; Li et al., 2007; Kang and Lowery, 2014). These effects are dissociable from effects on stimulated cell bodies, due to lower threshold of activation in fibers relative to cell bodies (Nowak and Bullier, 1998; Dostrovsky et al., 2000; McIntyre et al., 2004). Consequently, STN-DBS and STN-inactivation have distinct effects on activity throughout the basal ganglia (Creed et al., 2012), which is crucial, since coordinated activity in the basal ganglia network is necessary for driving reward seeking behavior (Sesack and Grace, 2010). Another consequence of network modulation by STN-DBS is that it induces striatal dopamine release (Benazzouz et al., 2000a; Bruet et al., 2001; Zhao et al., 2009; Shon et al., 2010; He et al., 2014), which is also not observed with STN lesion (Winter et al., 2008; Walker et al., 2009). Striatal dopamine release signals the difference between expected and experienced reward to drive reward learning, and invigorates action selection including reward seeking (For reviews, see Howe et al., 2013; Berke, 2018). By inappropriately elevating dopamine levels, STN-DBS could increase the noise in the dopamine-mediated reward-prediction error signal, or could induce long-term plasticity in the striatum which could also contribute to impairments in reward seeking behavior (Benazzouz et al., 2000a; Bruet et al., 2001; Gubellini et al., 2006; Carcenac et al., 2015).

### STN-DBS Potentially Induces Ectopic Stimulation

**Figure 8:**
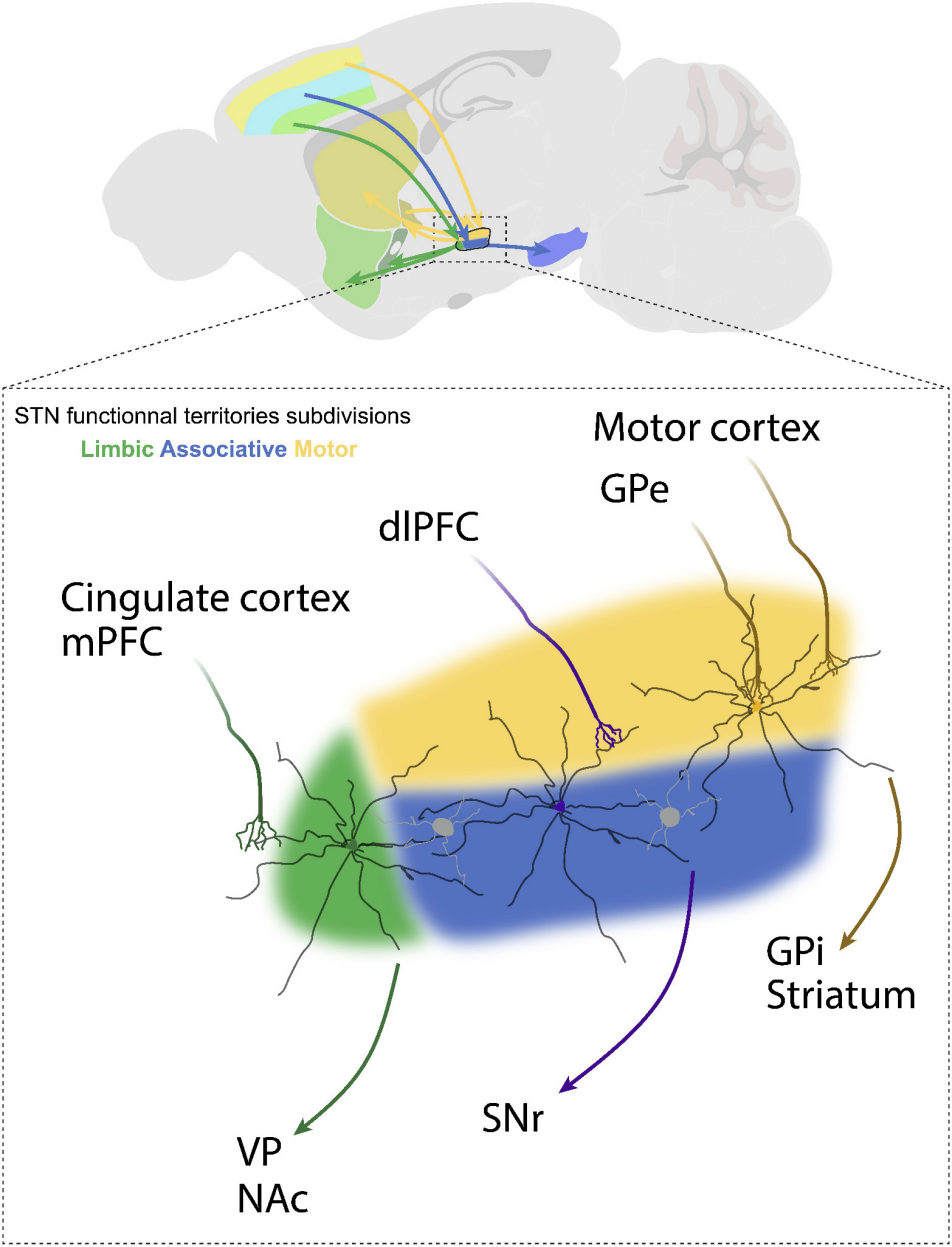
Afferent and efferent connections of STN functional subdivisions. The subthalamic nucleus (STN) is subdivided into a dorsolateral motor territory, a ventromedial associative territory, and a medial limbic territory. Each functional territory receives input from different cortical regions or the external segment of the globus pallidus (GPe), and in turn projects to different downstream structures, including the internal segment of the globus pallidus (GPi), substantia nigra pars reticulata (SNr), nucleus accumbens (NAc) and ventral pallidum (VP). These input-output interactions provide for parallel control of motor, cognitive, and emotional functions. The STN is composed of interneurons and glutamatergic projection neurons whose dendrites may arborize over a distance of up to 500 μm. This is important, because individual STN neurons may physically span into adjacent territories and be effected by DBS applied to these adjacent subdivisions.

An alternative hypothesis to explain the effects of STN-DBS on reward seeking is current spread outside the motor territory of the STN and potentially outside the STN itself. The STN can be divided into three functional territories (Figure 8): motor, associative and limbic, based on its afferent and efferent connections (Lambert et al., 2012; Hamani et al., 2017; Emmi et al., 2020). The caudal and dorsolateral part form the motor STN; it receives inputs from the primary motor cortex and GPe and projects to the GPi and the striatum (Benarroch, 2008). The associative STN lies in the ventral lateral aspect of the rostral nucleus, it receives input from the dorsolateral prefrontal cortex and innervates the SNr (Benarroch, 2008). Activity in the associative territory supports cognitive aspects of motor behavior, including impulsivity and attentional control (Frank, 2006; Alegre et al., 2013; Obeso et al., 2013). Finally, the rostromedial tip of the STN constitutes the limbic territory. This division receives inputs from the medial prefrontal and anterior cingulate cortices and projects to the ventral pallidum and the nucleus accumbens (Cavdar et al., 2018; Emmi et al., 2020). Limbic functions of the STN involve reward encoding and sensory integration to drive appropriate emotional states (Drapier et al., 2008; Lardeux et al., 2009; Eitan et al., 2013; Zenon et al., 2016). Therefore, stimulation of the limbic and/or associative territories is one possible explanation for the effects of STN-DBS on reward-related behavior (Mallet et al., 2007; Zoon et al., 2019). While these subdivisions are established in human and non-human primate, the well-defined topographic segregation is less clear in rodents (Alkemade et al., 2015). In rats, STN neurons with cell bodies localized in a given territory can extend dendrites across the length of the nucleus (Afsharpour, 1985). Thus, the current spread from STN-DBS electrodes, even if well-placed within the motor territory of the STN could also modulate STN neurons in non-motor territories (Figure 8). Beyond this, the STN is embedded within the zona incerta, and sits adjacent to the internal capsule and pallidofugal system (Hamani et al., 2004; Parent and Parent, 2004). These areas would be modulated by current spread outside the STN, and could be relevant for non-motor effects of STN-DBS. As mentioned above, in rodent studies, monopolar stimulation (which induces a larger current spread relative to bipolar stimulating electrodes) is associated with greater deficits in reward seeking (Figure 5). While the effects of monopolar vs. bipolar stimulation on induction of apathy or reward seeking behavior have not been directly compared in the clinic, there is evidence to suggest that high current amplitude and the use of monopolar electrodes are associated with worse psychiatric outcomes (Deli et al., 2011; Chopra et al., 2012). This is consistent with the hypothesis that reward-seeking deficits could be accounted for by current spread to limbic or associative territories of the STN, or to STN-adjacent nuclei and fiber tracts (Tan et al., 2013).

Even if it is current spread to STN subterritories and adjacent structures, and not modulation of the motor STN *per se* that drives reward-seeking deficits, this still does not explain the precise mechanisms underlying these deficits. For example, the reward-related effects could also be due to antidromic activation of afferent structures such as the prefrontal cortex (Irmen et al., 2020), or to modulation of downstream structures such as ventral pallidum or nucleus accumbens (Hahn et al., 2008; Cavdar et al., 2018). Likewise, STN-DBS modulates dopamine tone through polysynaptic outputs of the STN proper leading to stimulation of midbrain dopamine neurons, or through direct activation of dopamine fibers arising from current spread beyond the STN borders (Benazzouz et al., 2000a; Bruet et al., 2001; Tan et al., 2011, 2012; Carcenac et al., 2015). To disentangle these different possibilities, sophisticated approaches to circuit dissection, such as optogenetics, will be required.

## Determining the Neural Mechanisms Underlying the Effects of STN-DBS: Future Prospects with Optogenetics

Optogenetics refers to a suite of engineered ion channels that are activated by light in a specific wavelength, and flux ions in response to activation. Channelrhodopsin (ChR2) is non-selective cation channel, that when exposed to blue light (~473 nm), allows sodium and calcium to flow into the cell along their concentration gradients, thereby inducing depolarization and action potentials (Nagel et al., 2003). Conversely, the inhibitory halorhodopsin is a chloride pump that is activated upon stimulation with amber light (~590 nm), increases the intracellular chloride concentration, thereby hyperpolarizing the cell and inhibiting the firing action potentials (Zhang et al., 2007). The location of the injected virus expressing the opsin and placement of the optic fiber for light delivery allows for spatial control of neural activation, while cell-type or projection-specific control of neural populations can be achieved by expressing viruses using intersectional genetic strategies. Finally, the pattern of light stimulation allows for tight temporal control of neural activity (Liewald et al., 2008). Optogenetics has yielded highly valuable insight to functional connectivity and activity within intact or pathological circuits, and could be leveraged to resolve outstanding questions regarding STN-DBS mechanisms.

Optogenetics was first leveraged to elucidate the role of STN in motor processes over 10 years ago, when a seminal study by Gradinaru et al. (2009) targeted the STN with excitatory and inhibitory optogenetic approaches. This investigation first tested the hypothesis that the motor effects of STN-DBS were due to local inhibition. However, optogenetically silencing cell bodies of the STN by activation of halorhodopsin was unable to rescue motor deficits in a 6-OHDA model of PD. Instead, using ChR2 to selectively activate terminal fields of cortical afferents into the STN rescued unilateral motor deficits in the PD model, suggesting a critical role of antidromic activation of “hyperdirect” cortico-STN pathway in the motor effects of DBS (Li et al., 2007; Fraix et al., 2008). Optogenetic activation of STN cell bodies also did not rescue motor deficits, arguing that STN-DBS does not exert its effects through driving action potentials in efferent STN fibers. However, these experiments stimulated at 130 Hz, while kinetics of the variants of ChR2 available at the time were not able to follow such high stimulation frequencies (Gunaydin et al., 2010). More recent studies with mutated opsins [i.e., Chronos, which is capable of following frequencies over 100 Hz (Saran et al., 2018)] have suggested that activation of cell bodies at frequencies relevant to DBS may indeed rescue motor deficits in a PD model (Yu et al., 2020). As with DBS, these investigations demonstrate a frequency-dependence of optogenetic effects, and have elucidated multiple neural mechanisms driving the therapeutic motor effects of STN-DBS in animal models.

The neural mechanisms underlying the potential adverse psychiatric effects of STN-DBS have received considerably less attention (Pan et al., 2014; Yoon et al., 2014, 2016; Tian et al., 2018). In the future, stimulating the STN with fast opsins such as Chronos or ChETA (Gunaydin et al., 2010) in the context of motivation and reward-learning paradigms will provide unique insights about the causality of the STN itself for the effects of STN-DBS on reward-related behavior. A single report has suggested that optogenetic stimulation of STN cell bodies at frequencies >100 Hz can reduce the breakpoint for sucrose, and that this effect is critically dependent on stimulation frequency and pulse width (Tiran-Cappello et al., 2018). However, this report did not distinguish between STN subdivisions, and as discussed above, current spread to the limbic and/or associative STN territories is one prevailing hypothesis for decreased reward seeking following STN-DBS. This hypothesis could be tested by selectively manipulating those functional subterritories. Because of the small size of the STN in rodents such a spatial resolution could be achieved by targeting pathway-specific output structures. For example, a recent study investigated PD-related pain, by injecting ChR2 within the STN and placing optic fibers in different STN output structures, such as substantia nigra reticulata or ventral pallidum to target the motor and limbic STN subterritories, respectively (Luan et al., 2020). Or, akin to the work by Gradinaru et al. (2009), antidromic activation of afferents can be modeled by expressing excitatory opsin in STN-projecting structures and placing fibers above the STN (Sanders and Jaeger, 2016; Sanders, 2017). The medial prefrontal and anterior cingulate cortices constitute major limbic inputs to the STN; thus, manipulations of these pathways could yield important insight into the role of STN-DBS in the context of reward seeking.

The application of optogenetics has also shown promise for the development of novel deep brain stimulation protocols. In the clinic, and in all pre-clinical literature cited here, stimulation is applied at high frequencies (above 50 Hz, Figure 5). However, with optogenetics, specific cell types can be stimulated at precise physiological frequencies, and these physiological activity patterns can be used to drive plasticity within activated circuits. Proof of concept of this approach have been demonstrated for the motor symptoms of PD (Mastro et al., 2017) and for addiction (Pascoli et al., 2011, 2014; Creed et al., 2015; Creed M., 2018). In both of these applications, targeted stimulation of genetically-defined neural circuits was able to reverse behavioral impairments by selectively normalizing circuit function. While this strategy has not yet been demonstrated with STN stimulation, it is possible that selective activation of STN subdivisions at frequencies capable of driving long-term adaptations may induce persistent motor benefits without requiring continuous stimulation that carries with it the potential for adverse motivational effects.

In sum, sophisticated circuit dissection with optogenetics could be used to understand the role of the STN and its functional subterritories in coordinating adaptive motor and reward-related behaviors. With this insight, it may be possible to rationally stimulate the STN in order to achieve sustained motor benefits with lower risk of adverse effects on reward-related behavior. Conversely, it is possible that targeted manipulation of the associative or limbic territories of the STN could be leveraged to optimize or develop novel DBS paradigms to treat symptoms of addiction or obsessive compulsive disorder.

## Conclusion

STN-DBS has become a mainstay therapy for movement disorders, and has been proposed to be applied earlier in course of PD, and potentially expanded to other indications, such as obsessive and compulsive disorders or addiction. Clinically, side effects such as depression, impulsivity and apathy have been reported with STN-DBS, which presents a major therapeutic limitation. To prevent side-effects, we must understand their neural underpinnings. In this respect, pre-clinical models have the advantage of being able to dissociate the effects of STN-DBS *per se* from underlying disease pathology or confounding effects of dopaminergic medications. Here, we focus our review on the specific dimensions of reward motivation, seeking and consumption, which can be clearly defined in operant tasks. When limiting our review to this specific scope, we find that STN-DBS consistently decreases reward motivation, seeking and consumption across a variety of behavioral models. Interestingly, studies that lesioned or inactivated the STN showed no consistent effect on reward-related behavior. Moreover, monopolar stimulation and bilateral stimulation, which both increase the volume of tissue activated, tend to be associated with more severe reward seeking deficits. Together, these observations suggest that reward seeking deficits may not be mediated by local effects within the STN *per se*, but by modulation of afferent or efferent structures of the limbic territory of the STN, or by current spread to adjacent fiber tracts. To definitively address these questions, optogenetic tools could be used to dissect the STN circuitry and establish links of causality between DBS effects on STN microcircuitry and reward seeking deficits, as has been done for the motor domain of STN-DBS.

A final consideration is that, we focused our review on only the dimension of reward motivation, seeking and consumption in tasks without conflict. Impulsivity, which is another commonly reported effect of STN-DBS is beyond the scope of the current review. However, extensive evidence has implicated the STN in arresting behavior, particularly under conditions of conflict to allow more time to accrue for an optimal decision to be made in rodents (Baunez and Robbins, 1997, 1999b) and patients (Bastin et al., 2014; Benis et al., 2016). This was recently elegantly demonstrated using optogenetic modulation of the STN; STN activation was able to abruptly interrupt reward consumption, while STN-inhibition prevented the ability of novel, salient stimuli to abort reward consumption (Fife et al., 2017). In real-world contexts, reward-related behavior often occurs under conditions of conflict, or with costs associated to reward seeking or consumption. This is particularly relevant in the context of impulse control disorders or addictions, in which reward seeking becomes maladaptive because of its association with adverse consequences. Therefore, future directions for understanding the effect of STN-DBS on reward-related behavior in a translational context will require the application of decision-making tasks that capture dimensions of risk-reward balance, as well as cognitive and motor impulsivity. Understanding the mechanisms underlying the potential adverse psychiatric effects of STN-DBS, and disentangling these from the substrates underlying its beneficial motor effects will be necessary for optimizing its therapeutic potential.

## Data Availability Statement

The original contributions presented in the study are included in the article/supplementary materials, further inquiries can be directed to the corresponding author.

## Author Contributions

YV performed the literature review. YV and MC wrote the manuscript. Both authors contributed to the article and approved the submitted version.

## Conflict of Interest

The authors declare that the research was conducted in the absence of any commercial or financial relationships that could be construed as a potential conflict of interest.
